# Unanticipated difficult airway due to undiagnosed oropharyngeal stenosis: a case report

**DOI:** 10.1186/s40981-016-0032-y

**Published:** 2016-06-02

**Authors:** Mina Nishimori, Miyako Matsumoto, Hideki Nakagawa, Noriko Ichiishi

**Affiliations:** 1 Department of Anesthesiology, Seibo International Catholic Hospital, 2-5-1, Nakaochiai, Shinjuku, Tokyo, 161-8521 Japan; 2 Department of Anesthesiology, Nihon University School of Medicine, 30-1, Oyaguchikamicho, Itabashi, Tokyo, 173-8610 Japan; 3 Department of Otolaryngology, Seibo International Catholic Hospital, 2-5-1, Nakaochiai, Shinjuku, Tokyo, 161-8521 Japan

**Keywords:** Oropharyngeal stenosis, Difficult airway, Upper airway surgery

## Abstract

Unanticipated difficult airway is a challenging problem for anesthesiologists. Oropharyngeal stenosis (OPS) is a rare complication of upper airway surgery which may cause difficult airway. We present a patient whose postsurgical OPS was revealed during the induction of general anesthesia, and necessitated reschedule of surgery and tracheotomy. We also discuss the etiology and risk factors of postsurgical OPS.

## Background

An unexpected difficult airway during the induction of general anesthesia is a condition that is best avoided. A difficult intubation can occur because of anatomical abnormalities or situational factors such as airway inflammation. Anatomical factors indicative of difficult airway include high body mass index, older age, Mallampati grade III or IV, severely limited jaw protrusion, and thyromental distance of less than 6 cm [1]. However, even those predictors could fail at predicting difficult laryngoscope. We present a case of unexpected difficult intubation due to undiagnosed oropharyngeal stenosis (OPS).

## Case presentation

A 46-year-old 48 kg, 158 cm Chinese woman was scheduled for elective colectomy. Because she did not speak Japanese or English, we used a medical history questionnaire written in Chinese. She did not indicate any condition or surgical history. Although an interpreter (a Japanese nurse who spoke some Chinese) helped us with the pre-anesthesia visit, a detailed interview was unable. She was classified as Mallampati IV (uvula not visible), but we did not find other factors indicative of a difficult airway (BMI < 25, interincisal distance >6 cm, thyromental distance >6 cm, jaw protrusion not limited, neck flexion-extension >90 degree). A rapid induction and tracheal intubation using a direct laryngoscope was scheduled, with a fiberscope made readily available.

General anesthesia was induced with fentanyl 100mcg, propofol 100 mg and rocuronium 40 mg. Mask ventilation was easy. Under direct laryngoscopy (Macintosh #3), we could not observe the epiglottis because there was a membrane-like tissue in her pharynx with a narrow opening. When we examined her airway with a fiberscope, we could not observe the larynx through the opening, and we found the stenotic segment to be stiff and not expandable. We discussed the possibility of awakening the patient and performing a fibreoptic intubation because we believed that glossoptosis due to general anesthesia could have caused the larynx to be unobservable. However we decided to postpone the surgery for two reasons: firstly, the opening seemed to be too stiff and narrow for an endotracheal tube to pass through, and secondly, we had no information about the cause and etiology of her airway stenosis. Sugammadex 200 mg was administered, and spontaneous respiration was resumed within five minutes. She came out of anesthesia without difficulty breathing and was sent back to the ward.

**Fig. 1:**
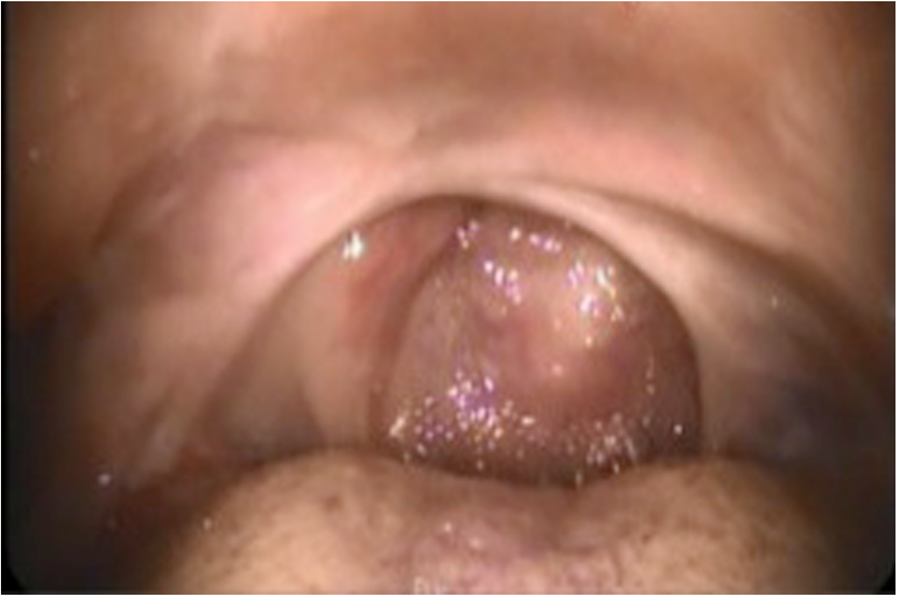
Laryngofiberscopic view of the oropharynx. Adhesion of the anterior tonsillar pillars and inferior tonsillar fossa to the tongue base is observed. Uvula is obliterated

**Fig. 2:**
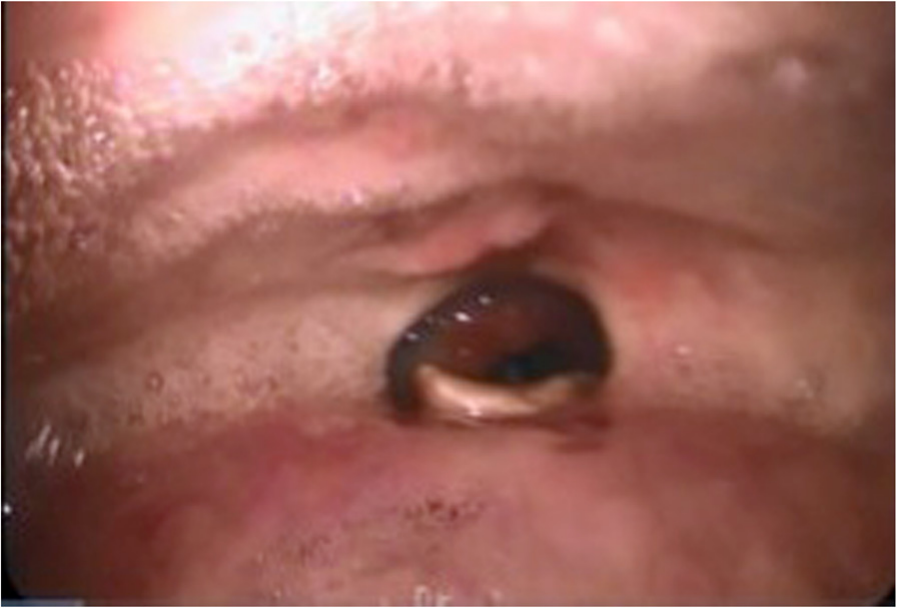
Laryngofiberscopic view of the cicatrix. The diameter was about 6 mm. Epiglottis is observed beyond the opening

**Fig. 3:**
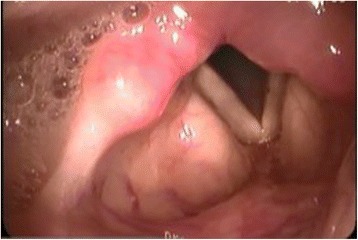
Laryngofiberscopic view beyond the cicatrix. The supraglottic space and the vestibule are intact

The next day, her airway was evaluated by an otolaryngologist. Laryngofiberscopy revealed an all-round fibrous cicatrix located between the tongue base and lateral-posterior wall of the pharynx obliterating the oropharynx, including the uvula (Fig. 1). The diameter of the opening of the cicatrix was about 6 mm (Fig. 2). With the patient awake, we could observe the larynx through the opening and found the supraglottic space and the vestibule to be intact (Fig. 3). We also hired a professional medical interpreter (a Chinese native speaker) to record her medical history in detail. It revealed that she undertook adeno-tonsillectomy under local anesthesia at the age of 23 in China, and since then she had been suffering from dysphagia (difficulty swallowing solid food). She had lived on softened food and had adapted well to this problem. Since she did not consider this a serious problem and did not experience any other symptoms such as speech problems and difficulty breathing, she did not tell us about it on our preoperative visit.

It was possible to insert a 3 mm fiberscope through the opening of the cicatrix and through the vocal code; awake-fibreoptic intubation seemed to be possible. However, the difficulty of respiratory management using a tracheal tube of less than 6 mm of outer diameter (which means an inner diameter of 4 to 4.5 mm) and the postoperative possible upper airway obstruction due to inflammation or scarring of the narrow opening, led to our decision to secure the airway by a tracheotomy. After the tracheotomy under local anesthesia, general anesthesia was induced and surgery was performed uneventfully.

## Discussion

Oropharyngeal stenosis is caused by adhesion of the anterior pillars and inferior tonsillar fossa to the tongue base. Symptoms may include dyspnea on exertion, dysphagia, poor weight gain, and obstructive sleep apnea. Some patients may be asymptomatic [2]. Before 1940, most cases were due to infection, mainly pharyngeal syphilis [3]. After 1940, the most common etiology of OPS has been reported to be surgical trauma associated with adeno-tonsillectomy [2]. Risk factors for developing OPS after adeno-tonsillectomy include excessive cautery, postoperative bleeding and infection, and keloid tendency [2], but the prevalence has been reported to be very small [2]. This case was thought to be a rare case of OPS caused by adeno-tonsillectomy. Other causes include airway burn and systemic inflammatory diseases such as sarcoidosis [4] and Behçet’s syndrome [5].

However, recent studies report higher prevalence of postoperative OPS among the patients who underwent more complicated upper-airway surgery such as pharyngoplasty for obstructive sleep apnea (OSA) [6, 7]. Prager et al. indicated the prevalence of postoperative OPS to be 8.2 % in their retrospective chart review of 104 pediatric patients who underwent multilevel upper airway surgery including lingual tonsillectomy [8]. Cohen et al. reviewed their 65 adult patients who underwent surgery for OSA. Among the patients who underwent UPPP (uvulopalatopharyngoplasty) or TORS (trans-oral robotic surgery) including tongue-base resection, the prevalence of postoperative OPS was 7.8 % [9]. In both reports, the authors indicate a single-stage procedure including tongue-base resection as a risk factor, along with the aforementioned risk factors of postoperative OPS [8, 9].

Several case series of post-surgical OPS are written by otolaryngologists [2, 3, 8, 9]. One of them briefly commented on unexpected difficult airway during the induction of general anesthesia [2], but most of them did not. Although there is a case report of unanticipated difficult intubation due to an undiagnosed pharyngeal stenosis [10], this complication is still not widely recognized by anesthesiologists.

Our case raises two issues into discussion; the lack of preoperative interview and the awareness of postoperative OPS can cause unexpected difficult airway. For this particular case, there was a serious communication problem. Due to the language barrier, we were unable to obtain the information about the patient’s history of adeno-tonsillectomy and subsequent dysphagia, because the patient considered it as a trivial problem. Ideally, medical interpreters should always be available, but this was not the case for our facility, as well as for many others. With the increase of patients unable to speak Japanese, this issue is becoming more common. In addition, even if we had no language issues and were aware of her surgical history, we could have overlooked her OPS because it is a very rare complication of adeno-tonsillectomy. However, considering that we may have more chance to encounter postsurgical OPS in the future with the development of complicated upper-airway surgery, we should be able to recognize and anticipate this complication. In our pre-operative interview, we should specifically ask patients about their history of upper-airway surgery. If the patient has had surgery, an evaluation through detailed interview and close observation should be performed. We should also consult an otolaryngologist if we suspect a complication. Laryngofiberscopy will provide us with useful information for planning how to secure the patient’s airway.

## Conclusions

We reported a case of preoperatively undiagnosed OPS which resulted in unexpected difficult laryngoscope that necessitated a reschedule of surgery. Two factors prevented us from foreseeing the patient’s OPS: insufficient communication due to language barrier and the patient’s dismissal of dysphagia (her only problem due to OPS) as a harmless condition. We discussed the etiology of acquired OPS and we suggest that anesthesiologists be aware of this rare complication that could be on the rise.

This case was presented on September 5^th^, 2015, at the 55^th^ Annual Meeting of JSA Kanto-Tokyo Region.

## Consent to publish

Written informed consent was obtained from the patient for publication of this case report and any accompanying images. A copy of the written consent is available for review by the editor-in-chief of this journal.

## Abbreviations

OPS: oropharyngeal stenosis
OSA: obstructive sleep apnea
TORS: trans-oral robotic surgery
UPPP: uvulopalatopharyngoplasty
